# Dental College of the University of Michigan

**Published:** 1888-07

**Authors:** 


### Dental College of the University of Michigan.

The thirteenth annual commencement exercises of the Dental College of the University of Michigan were held in University Hall Thursday, June 28th, 1888. The degree of D.D.S. was conferred upon the following named persons:

| NAME. | RESIDENCE. |
|---|---|
| Horace Albert Benson.. | Ohio. |
| Clarence Walker Berry.. | Michigan. |
| Wm. Townsend Binzley. | Pennsylvania. |
| Harriette P. Brierley.... | England. |
| Elwyn Butts ........ | Iowa. |
| Rollin Edward Drake... | Michigan. |
| William Fraser Dunlop.. | Michigan. |
| Frank Howard Essing. | Michigan. |
| Willam Burton Flynn... | Michigan. |
| Sherman M. Fowler..... | Michigan. |
| Jeromino Jill Garcia.... | U. S. of C. |
| Arthur Newton Hart.... | Michigan. |
| Elmer Bertrand Hause.. | Michigan. |
| Oliver Wendell Huff. .. | Kansas. |
| Egbert T. Loeffler.. .... | Michigan. |
| Otto Marx ........ | Ohio. |
| Thoma Stuart Maxwell. | Wisconsin. |
| Charles E. Meerhoff.. .. | Indiana. |
| Richard Edward Moll... | Michigan. |
| Irvin Myers.................. | Michigan. |
| Rudolph Paul Nagle........ | Wisconsin. |
| Harry Cox Nickels......... | Michigan. |
| Charles Walter Nutting..... | Minnesota. |
| Homer Ellsworth Parshall.. | Michigan. |
| William Orlando Randall... | Michigan. |
| Henry Charles Raymond. .. | England. |
| Theckla Ste n Reuter........ | Wisconsin. |
| Henry William Riser....... | Iowa. |
| Martha Josephene Robinson | Ohio. |
| Henry Martin Seybold ..... | Michigan. |
| Michael Cornelius Sheehan.. | Michigan. |
| Lucius Chipman Smith..... | Michigan. |
| Sherman M. Stauffer..... | Illnois. |
| Martin Dogener Vandenberg | Michigan. |
| Alfred Frederick Webster.. | Ontario. |
| William Holt Woodburn... | Scotland. |
| Walter Thomas Wright..... | Michigan. |

